# Clonal Structure and Characterization of *Staphylococcus aureus* Strains from Invasive Infections in Paediatric Patients from South Poland: Association between Age, *spa* Types, Clonal Complexes, and Genetic Markers

**DOI:** 10.1371/journal.pone.0151937

**Published:** 2016-03-18

**Authors:** Weronika M. Ilczyszyn, Artur J. Sabat, Viktoria Akkerboom, Anna Szkarlat, Joanna Klepacka, Iwona Sowa-Sierant, Barbara Wasik, Maja Kosecka-Strojek, Aneta Buda, Jacek Miedzobrodzki, Alexander W. Friedrich

**Affiliations:** 1 Department of Microbiology, Faculty of Biochemistry, Biophysics and Biotechnology, Jagiellonian University, Krakow, Poland; 2 Department of Medical Microbiology, University of Groningen, University Medical Center Groningen, Groningen, The Netherlands; 3 Department of Clinical Microbiology, Children’s University Hospital, Jagiellonian University, Krakow, Poland; Rockefeller University, UNITED STATES

## Abstract

The aim of current study was to examine clonal structure and genetic profile of invasive *Staphylococcus aureus* isolates recovered from infants and children treated at the Jagiellonian University Children’s Hospital of Krakow, Poland. The 107 invasive *S*. *aureus* isolates, collected between February 2012 and August 2014, were analysed retrospectively. Antimicrobial susceptibility testing, *spa* typing and DNA microarray analysis were performed to determine clonal distribution, diversity and gene content in regard to patients characteristics. In total, 107 isolates were recovered from 88 patients with clinical symptoms of invasive bacterial infection. The final set of 92 non-duplicate samples included 38 MRSA isolates. Additionally, a set of 54 *S*. *aureus* isolates collected during epidemiological screening was genotyped and analysed. There were 72 healthcare-associated (HCA) and 20 community-onset (CO) infection events caused by 33 and 5 MRSA isolates, respectively. The majority of isolates were affiliated with the major European clonal complexes CC5 (t003, *spa*-CC 002), CC45 (*spa*-CC 015), CC7 or CC15 (t084, t091, *spa*-CC 084). Two epidemic clones (CC5-MRSA-II or CC45-MRSA-IV) dominated among MRSA isolates, while MSSA population contained 15 different CCs. The epidemiological screening isolates belonged to similar genetic lineages as those collected from invasive infection cases. The HCA infection events, *spa* types t003, t2642 or CC5 were significantly associated with infections occurring in neonates and children under 5 years of age. Moreover, carriage of several genetic markers, including *erm(A)*, *sea (N315)*, *egc-*cluster, *chp* was significantly higher in isolates obtained from children in this age group. The *spa* types t091 and t008 were underrepresented among patients aged 5 years or younger, whereas *spa* type t008, CC8 and presence of *splE* was associated with infection in children aged 10 years or older. The HCA-MRSA strains were most frequently found in children under 5 years, although the majority of invasive infections was associated with MSSA strains. Moreover, an association between age group of children from the study population and a specific strain genotype (*spa* type, clonal complex or genetic content) was observed among the patients.

## Introduction

*Staphylococcus aureus* is one of major human pathogens, associated with wide spectrum of localized or systemic infections, including bacteremia or sepsis. In younger patients, neonates and children, the prolonged hospitalization, antibiotic exposure, invasive procedures and devices have been indicated to increase risk of infection with multi-resistant pathogens [1]. According to the Polish Neonatology Surveillance Network report, between the years 2009-2012, *S*. *aureus* was responsible for 6.5% of infections in newborns. Approximately 33% of those events was caused by methicillin resistant *S*. *aureus* (MRSA) [2]. In United Kingdom, *S*. *aureus* was reported as a common cause of late onset of neonatal sepsis, and was identified in 13% of bacterial isolates from blood cultures of neonates aged from 0 to 28 days [1]. Similarly, in Sweden, *S*. *aureus* was the most common pathogen found in blood samples from children undergoing infection, irrespectively of underlying risk factors [3]. Although, both MRSA and methicillin susceptible (MSSA) *S*. *aureus* strains are responsible for only 1% of all-cause bacteremia and meningitis in infants, observed mortality rates are high and amount to 26% and 24%, respectively [1]. The severity and outcome of infection depend strongly on the virulence repertoire of invasive strain and immune system status of the host. Especially, the immunocompromised patients have an increased risk of *S*. *aureus* colonization, potentially followed by infection, further morbidity and unfavourable outcome. Risk of acquisition of multi-resistant pathogens can be elevated by preterm birth, very low birth weight, frequent or long-term admissions, need for antimicrobial therapy, presence of comorbidities or/and immune dysfunction [3–5]. Among children with malignancy, congenital heart disease or liver transplant recipients, *S*. *aureus* accounts for up for 9–13%, 13% and 20% of blood stream infections, respectively [4,6–8]. The effective prevention is greatly impeded, as *S*. *aureus* is ubiquitous in the environment and its asymptomatic carriage is more a rule than an exception. Moreover, treatment of *S*. *aureus* infections is challenging due to its multi-resistant profile and ability to produce a wide range of virulence factors, including staphylococcal enterotoxins, proteases, leukocidins, proteins associated with immune-evasion or adhesion [9,10]. Many epidemiological investigations have been focused on *S*. *aureus* strains recovered from blood specimens or invasive infections, but only a few have studied strains collected from paediatric patients [1,11]. The aim of current study was to examine clonal structure of isolates recovered from invasive infections in infants and children treated at the Jagiellonian University Children’s Hospital of Krakow, Poland. Moreover, emergence and distribution of *S*. *aureus* genotypes were analysed, and supplemented with results from typing of isolates from epidemiological screenings. Utilization of *spa* typing and DNA microarrays (StaphyType, Alere Technologies) allowed for molecular characterization of *S*. *aureus*, including resistance and virulence markers profiling. Moreover, the relationships between age and epidemiological classification of infection event, resistance profile, corresponding strain *spa* type or clonal complex (CC), were investigated.

## Materials and Methods

### Hospital characteristics

The study was conducted at the Jagiellonian University Children’s Hospital (UCH): a 529-bed, tertiary care referral clinic and academic institution located in Krakow metropolitan area, in Malopolska region of South Poland. UCH is a highly specialized reference center for most severely ill paediatric patients including children with neoplastic diseases, neonatal heart defects, burns, congenital defects, neonates with low birth weight or undergoing bone marrow transplants. Annually UCH holds app. 36 000 admissions and 150 000 outpatients from all over Poland and abroad, with the majority originating from Malopolska, Silesian or Subcarpatian regions. Therefore, UCH provides medical and surgical care for children born in population of approximately 10 mln people and 45 360 km^2^ area, comparable to Slovakia or other medium-sized European country. Included 88 patients originated from over 50 cities of South Poland and were treated in thirteen departments in UCH: Pathology and Neonatal Intensive Care (IC) Unit (n = 22, mean age 0.1 year, approximately 40 days), Nutritional Therapy (n = 11, mean age 2.7 years), Anaesthesiology and IC Unit (n = 11, mean age 5.2 years), Neurosurgery (n = 10, mean age 2.6 years), Rehabilitation (n = 7, mean age 7.0 years), Surgery (n = 8, mean age 0.7 year, approximately 7 months), Haematology and Oncology (n = 7, mean age 8.7 years), Cardiac Surgery and Cardiac IC (n = 3, mean age 0.2 year, approximately 2 months), Cardiology (n = 2, mean age 0.8 year, approximately 10 months), Gastroenterology (n = 2, mean age 3.4 years), IC (n = 2, mean age 12.7 years), Rheumatology and Environmental Diseases (n = 2, mean age 10.6 years), and Transplantology (n = 1, age 4.5 years). Moreover, a set of epidemiological screening isolates was collected from none-invasive infection patients, medical personnel, hospital spaces (e.g. nurse station, operating room) or equipment. The study was approved by Jagiellonian University and UCH Committee, as this was a retrospective study focused on bacteria characterization and did not require any patient involvement.

### Definitions

An invasive infection was defined as localized or systemic inflammatory response to the presence of *S*. *aureus* at otherwise sterile anatomical sites as blood, cerebrospinal fluid (CSF) or dialysis fluid (DF). Infection events were classified as community-onset (CO) when the positive culture was obtained from patients within the first 48 h of admission. The healthcare-associated (HCA) infection has been defined when symptoms developed 48 h after admission or within this period if the patient fulfilled any of the following criteria: i) patient have been hospitalized previously in UCH within the study period; ii) patient have been transferred to UCH from other healthcare facility; iii) patient have been treated in Department of Nutritional Therapy due to requirement of total parenteral nutrition and long-term presence of central venous catheter. The term infant was applied to young children under 1 year of age. The neonate refers to an infant in the first 1 month (30 days) of age. The mortality was the all-cause mortality [12,13].

### Bacterial isolates

From February 2012 to August 2014, 107 *S*. *aureus* isolates from invasive infection episodes detected and treated in UCH were recovered for this study. All isolates were collected from patients who developed clinical symptoms such as fever, leukocytosis, high level of procalcitonin or C-reactive protein on admission or during hospitalization. Clinical specimens, including blood (83.2%), CSF (14.0%) or DF (2.8%) were collected. Additionally, epidemiological screening swabs of *S*. *aureus* isolates were collected in three UCH departments (Cardiac Surgery and Cardiac IC n = 18; Nutritional Therapy n = 25; Pathology and Neonatal IC, n = 11) between September and November 2013. Overall 54 isolates were collected from 7 patients (mostly nasal swabs, n = 7), 32 members of medical personnel (nasal swabs or/and hand smears on agar substrate, n = 36) or environment (swabs of equipment, toys, hospital spaces as nurse stations or air probes, n = 11). Samples were inoculated into media optimized for detection of common paediatric pathogens. The BACTEC Peds Plus/F is culture medium that accommodates small-volume samples (≤3 ml of blood) and includes resins for antibiotic neutralization. Incubation with continuous monitoring and automatic testing was performed in Bactec FX system (Becton Dicknson, Franklin Lakes, NJ, USA). Positive samples were cultured on blood agar plates containing 5% sheep blood (20–24 h at 37°C). The *S*. *aureus* identification was performed by routine microbiology methods and VITEK^®^ 2 automated system (bioMérieux, Marcy l'Etoile, France). The isolates were further stored in tryptic soy broth with 10% glycerol at -80°C before re-culturing for purpose of current study.

### DNA extraction

For genomic DNA preparation, the *S*. *aureus* isolates were grown overnight (18–20 h at 37°C) on blood agar plates. Several colonies (full inoculation loop) were homogenized with a TissueLyzer II (Qiagen, Hilden, Germany), and genomic DNA was extracted using DNeasy Blood and Tissue Kit (Qiagen, Hilden, Germany) according to manufacturer’s instructions.

### Susceptibility testing

Susceptibility testing was carried out according to EUCAST recommendations [14]. MRSA isolates were identified using cefoxitin disks (30 μg) (Oxoid Ltd., Cambridge, UK), which was further confirmed by the detection of *mecA* gene in the DNA microarray analysis. The inducible macrolide-lincosamide-streptogramin B (MLS_B_) resistance was detected by disk diffusion method with use of clindamycin disks (2 μg) and erythromycin disks (15 μg) set 15–26 mm apart on Mueller-Hinton agar inoculated with *S*. *aureus* bacterial suspension (turbidity adjusted to 0.5 McFarland standard). The inducible phenotype causes a D-shaped inhibition zone around the clindamycin disk, blunted from the side of erythromycin disk. In the case of efflux mechanism of resistance, the entire zone of inhibition surrounding clindamycin disk is round. Other antibiotics tested *via* disc-diffusion method included: gentamicin (10 μg), linezolid (30 μg) or netilmicin (30 μg). Teicoplanin and vancomycin susceptibilities were determined by using E-test strips (bioMérieux, Marcy l'Etoile, France) according to the manufacturer’s instructions.

### *spa* typing

The *spa* typing, based on amplification of the variable X region of protein A gene, was performed as described previously [15]. The *spa* types were assigned using the Ridom StaphType software version 1.4 (Ridom GmbH, Würzburg, Germany) and the Ridom SpaServer (http://www.spaserver.ridom.de). The based upon repeat pattern (BURP) algorithm was used to calculate *spa* clonal complexes (*spa*-CCs) with following parameters: no exclusion regarding number of repeats; cost less or equal to 4; a cluster composed of 2 or more related *spa* types was regarded as CC; a *spa* type that was not grouped into a CC was considered a singleton.

### DNA microarray-based genotyping

The StaphyType system (StaphyType, Alere Technologies, Jena, Germany) is a microarray assay for diagnostic testing and/or epidemiological investigations. Briefly, the StaphyType kit allows for simultaneous detection of 334 *S*. *aureus* target sequences, including approximately 170 distinct genes and their allelic variants. The DNA microarray procedures were carried out according to the manufacturer's instructions. The assignment of strains to CCs or sequence types (STs) was performed automatically by StaphyType software [16]. In order to visualize strain relatedness, the obtained hybridization patterns were analysed using Splits Tree 4 software (www.splitstree.org) on default setting. Results for every single gene were converted into a code marked such: A = positive, T = negative, C = ambiguous, and used for construction of unrooted phylogenetic network [17].

### Statistical analysis

Differences of proportions in particular categorical data were assessed using Fisher’s exact test. The p-values <0.05 were considered as statistically significant.

## Results

### Patients and isolates characteristics

The collection comprised 107 *S*. *aureus* isolates recovered from 88 paediatric patients with symptoms of invasive infection and additionally 54 epidemiological screening isolates. Complete isolate information is provided in S1 Table. One isolate per patient was collected in the majority of invasive infection cases (73/88). Two isolates were obtained from each of thirteen patients, and two patients were identified with four isolates each. As summarized in Table 1, only first episode of *S*. *aureus* infection was considered for analysis in these 15 ‘multiple infection’ cases, with the exception of patients who experienced two separate episodes of infection with genetically different strains i.e. characterized by different *spa* types and/or affiliated with different CCs/SCC*mec* types. Therefore, the clonal structure and statistical analysis were based on a set of 92 non-duplicate invasive *S*. *aureus* isolates obtained from 88 patients, as summarized in detail in S2 Table.

**Table 1:** Details of 15 paediatric patients from whom between 1 to 4 *S*. *aureus* isolates were collected at UCH during the study period.

Isolate	Patient code^a^	Age	Isolation order	Difference between isolations [days]	Ward	Medium	*spa* type	*spa*-CC	DNA microarray CC
**PL274**	AF	10.5 y	1^st^	-	NT	blood	**t913**	*spa-*CC 012	CC30-MSSA
PL304		11.1 y	2^nd^	233	G	blood			
**PL322**	AT	3.1 y	1^st^	-	NT	blood	**t031**	*spa-*CC 015	CC45-MSSA
**PL209**		3.5 y	2^nd^	146	NT	blood	**t091**	*spa-*CC 084	CC7-MSSA
**PL291**	AX	1.6 y	1^st^	-	N	blood	**t056**	*spa-*CC 056 (no founder)	CC101-MSSA
PL296		1.7 y	2^nd^	36	N	blood			
**PL303**	AY	1.4 y	1^st^	-	R	blood	**t091**	*spa-*CC 084	CC7-MRSA-IV
PL312		1.6 y	2^nd^	93	R	DF			
PL347		2.7 y	3^rd^	386 (479 from 1^st^)	R	DF			
**PL349**		2.7 y	4^th^	11 (490 from 1^st^)	R	DF	**t003**	*spa-*CC 002	ST5/ST225-MRSA-II, Rhine-Hesse EMRSA
**PL271**	BF	1 m	1^st^	-	CIC	blood	**t015**	*spa-*CC 015	CC45-MRSA-IV, Berlin EMRSA
PL272		1 m	2^nd^	2	CIC	blood			
**PL279**	BL	28 d	1^st^	-	PN	blood	**t003**	*spa-*CC 002	ST5/ST225-MRSA-II, Rhine-Hesse EMRSA
PL280		28 d	2^nd^	0	PN	CSF			
PL281		1 m	3^rd^	5	PN	CSF			
PL283		1 m	4^th^	3 (8 from 1^st^)	PN	blood			
**PL287**	BR	6 d	1^st^	-	PN	blood	**t283**	singleton	CC5-MRSA-IV, Paediatric clone [*sed/j/r+]*
PL288		8 d	2^nd^	2	PN	blood			
**PL297**	BS	2 m	1^st^	-	NT	blood	**t015**	*spa-*CC 015	CC45-MRSA-IV, Berlin EMRSA
**PL205**		1.1 y	2^nd^	313	NT	blood	**t037**	*spa-*CC 012	CC30-MSSA
**PL236**	CA	1.9 y	1^st^	-	NT	blood	**t084**	*spa-*CC 084	CC15-MSSA
PL348		2.0 y	2^nd^	39	NT	blood			
**PL208**	CG	1.2 y	1^st^	-	NT	blood	**t124**	singleton	CC45-MSSA
PL357		1.8 y	2^nd^	226	NT	blood			
**PL250**	CN	28 d	1^st^	-	PN	blood	**t091**	*spa-*CC 084	CC7-MSSA
PL269		28 d	2^nd^	0	PN	CSF			
**PL334**	CO	4 m	1^st^	-	PN	CSF	**t2642**	*spa-*CC 2642	CC45-MRSA-IV, Berlin EMRSA
PL336		5 m	2^nd^	30	PN	blood			
**PL327**	CQ	3 m	1^st^	-	S	blood	**t127**	singleton	CC1-MSSA
PL339		7 m	2^nd^	100	G	blood			
**PL335**	CV	7 d	1^st^	-	PN	blood	**t14391**	singleton	CC398-MSSA
**PL238**		28 d	2^nd^	21	PN	blood	**t003**	*spa-*CC 002	ST5/ST225-MRSA-II, Rhine-Hesse EMRSA
**PL355**	CZ	4 m	1^st^	-	N	blood	**t005**	singleton	CC22-MSSA
PL356		4 m	2^nd^	1	N	CSF			

The *S*. *aureus* isolates selected for statistical analysis are bolded.

^a^To protect patients’ identity an alphabetic code has been assigned to each patient which had no correlation with patient’s names or initials.

y—year; m—month; d—day; NT—nutritional therapy, G—gastroenterology; N—neurosurgery; R—rehabilitation; CIC—cardiac surgery and cardiac intensive care; PN—pathology and neonatal intensive care; S—surgery; CSF—cerebrospinal fluid; DF—dialysis fluid

The median age of patients at the time of testing was 0.8 year (range, 5 days to 17.7 year). Almost half of the isolates (n = 42, 45.7%) was collected from infants under 6 months of age, including 21 neonates (22,8%). Male patients prevailed in analysed population (60.2%).

#### Healthcare-associated infections dominated in children age under 5 years

Among the 92 non-duplicate *S*. *aureus* isolates, 72 invasive infection episodes were classified as HCA (78.3%) and 20 episodes as CO (21.3%). Based on observed frequencies of infection events in the current study, patients under 5 years were significantly predisposed for HCA infection, while older children were associated with CO infection as presented in Table 2. The MRSA isolates accounted for 45.8% and 25.0% of HCA (33/72) and CO events (5/20), respectively. Similarly, considering associations observed for infection events, the HCA-MRSA isolates were significantly more often recovered from neonates and children under 5 years, while CO-MSSA in older patients (Table 2).

**Table 2:** Identified statistically significant associations between age of patients and infection event classification/UCH departments/HA MRSA/CO MSSA/ MRSA/MLS_B_/*spa* type/*spa-*CC/CC.

Groups	Subgroups	Age groups						
		<1 month	<6 months	<1 year	<3 years	<5 years	>5 years	>10 years
		N = 21	N = 42	N = 49	N = 64	N = 70	N = 22	N = 14
**HCA**^a^		n = 21 ↑	n = 39 ↑	n = 45 ↑	n = 58 ↑	n = 62 ↑	n = 10 ↓	n = 7 ↓
**N = 72**		p = 0.005	p = 0.002	p = 0.0009	p = 0.00004	p = 0.00008	p = 0.00008	p = 0.01
**CO**^b^		n = 0 ↑	n = 3 ↑	n = 4 ↑	n = 6 ↑	n = 8 ↑	n = 12 ↑	n = 7 ↑
**N = 20**		p = 0.005	p = 0.002	p = 0.0009	p = 0.00004	p = 0.00008	p = 0.00008	p = 0.01
**Pathology and Neonatal IC**		n = 17 ↑	n = 22 ↑	n = 23 ↑	n = 23 ↑	n = 23 ↑	n = 0 ↓	n = 0 ↓
**N = 23**		p = 0.0001	p = 0.0001	p = 0.0001	p = 0.0001	p = 001	p = 001	p = 0.02
**Nutritional Therapy**		n = 0 ↓	n = 1 ↓	n = 3 ↓				
**N = 13**		p = 0.03	p = 0.003	p = 0.03				
**Rehabilitation**				n = 1 ↓		n = 3 ↓	n = 5 ↑	
**N = 8**				p = 0.02		p = 0.02	p = 0.02	
**Haematology and Oncology**				n = 0 ↓	n = 0 ↓	n = 1 ↓	n = 6 ↑	
**N = 7**				p = 0.004	p = 0.0001	p = 0.0006	p = 0.0006	
**HCA MRSA**		n = 15 ↑	n = 25 ↑	n = 26 ↑	n = 31 ↑	n = 31 ↑	n = 2	
**N = 33**		p = 0.0002	p = 0.00002	p = 0.0004	p = 0.0001	p = 0.002	p = 0.002	
**CO MSSA**		n = 0 ↓	n = 2 ↓	n = 2 ↓	n = 4 ↓	n = 5 ↓	n = 10 ↑	n = 5 ↑
**N = 15**		p = 0.02	p = 0.009	p = 0.001	p = 0.0002	p = 0.0001	p = 0.0001	p = 0.048
**MLS**_**B**_		n = 15 ↑	n = 23 ↑	n = 26 ↑	n = 29 ↑			
**N = 34**		p = 0.0005	p = 0.002	p = 0.001	p = 0.02			
**MRSA**		n = 15 ↑	n = 26 ↑	n = 28 ↑	n = 33 ↑	n = 34 ↓	n = 4 ↓	
**N = 38**		p = 0.002	p = 0.0003	p = 0.001	p = 0.003	p = 0.01	p = 0.01	
**MRSA and MLS**_**B**_		n = 12 ↑	n = 20 ↑	n = 21 ↑	n = 23 ↑	n = 23 ↑	n = 2 ↓	
**N = 25**		p = 0.001	p = 0.0001	p = 0.0003	p = 0.004	p = 0.03	p = 0.03	
***spa* types**	**t003**	n = 10 ↑	n = 12 ↑	n = 13 ↑	n = 14 ↑	n = 14 ↑	n = 0 ↓	n = 3 ↑
	**N = 14**	p = 0.00003	p = 0.001	p = 0.001	p = 0.004	p = 0.02	p = 0.02	p = 0.01
	**t008**					n = 1 ↓	n = 3 ↑	
	**N = 4**					p = 0.04	p = 0.04	
	**t091**		n = 1 ↓					
	**N = 10**		p = 0.02					
	**t2642**		n = 5 ↑					
	**N = 5**		p = 0.02					
***spa-*CCs**	***spa-*CC 002**	n = 10 ↑	n = 14 ↑	n = 17 ↑	n = 18 ↑	n = 19 ↑	n = 0 ↓	
	**N = 19**	p = 0.001	p = 0.009	p = 0.0005	p = 0.01	p = 0.005	p = 0.005	
	***spa-*CC 008**							n = 3 ↑
	**N = 5**							p = 0.02
	***spa-*CC 084**		n = 4 ↓					
	**N = 20**		p = 0.01					
**CCs**	**CC5**	n = 12 ↑	n = 17 ↑	n = 20 ↑	n = 22 ↑	n = 23 ↑	n = 0 ↓	n = 0 ↓
	**N = 23**	p = 0.0003	p = 0.003	p = 0.0002	p = 0.001	p = 0.001	p = 0.001	p = 0.02
	**CC8**							n = 3 ↑
	**N = 6**							p = 0.04

The ↑ and ↓ symbols indicate that given category is over and underrepresented in analysed age group, respectively.

HCA—health-care associated; CO—community onset; CC—clonal complex; IC—Intensive Care; MRSA—methicillin resistant *S*. *aureus*; MSSA—methicillin susceptible *S*. *aureus*; MLS_B_−macrolide-lincosamide-streptogramin B (resistance)

^a^HCA group comprised of 33 MRSA and 39 MSSA isolates.

^b^CO group comprised of 5 MRSA and 15 MSSA isolates.

#### Mortality

During the study period, the 14 day all-cause mortality was 3.4% (3/88) and overall mortality equalled 4.5% (4/88). No statistically significant association was found between fatal outcome and characteristics of strains recovered from those four patients.

### Antimicrobial susceptibility testing

Among all tested isolates thirty-eight (41.3%) were MRSA positive. As summarized in Table 2, infants within first 6 months or 1 year after birth were significantly predisposed for infection by MRSA strains. Moreover, MRSA strains were rarely isolated from children aged 5 years or older. The MLS_B_ resistance was detected in 34 (37.0%) isolates. The association between MLS_B_ resistance and invasive infection was inversely proportional to the age of patients (Table 2). Statistical significance was detected in children under 3 years, under 1 year, and was most significant in neonates. The twenty-five isolates classified as both MRSA and MLS_B_, were mainly recovered from children under 5 years of age, and mostly overrepresented among isolates causing infection in infants. As presented in S1 Table, all tested isolates were sensitive to linezolid, gentamycin and vancomycin and only one was teicoplanin resistant (MIC 4.0 μg/ml).

### *spa* typing

**Fig 1:**
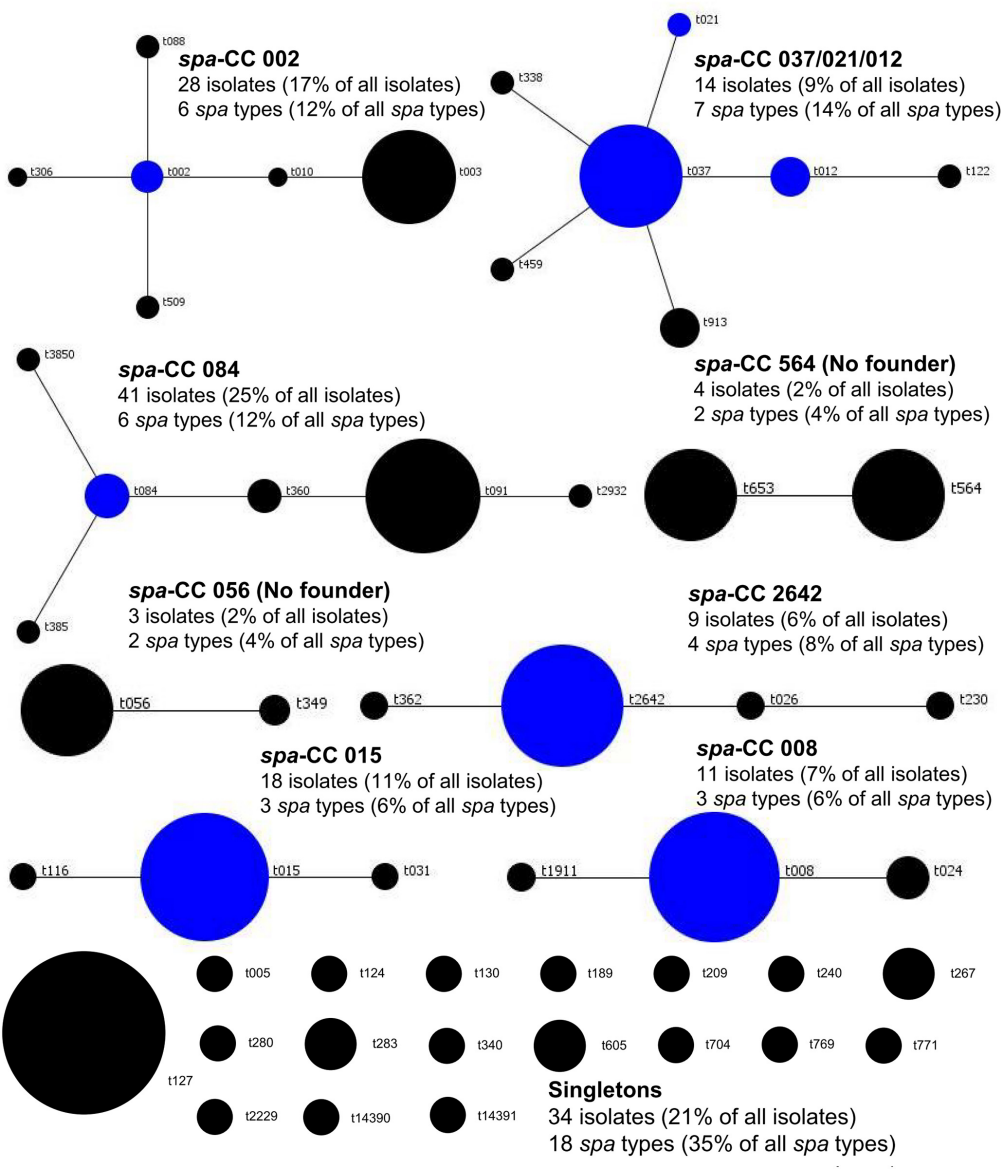
Population structure of 161 *S*. *aureus* isolates, including 107 invasive and 54 epidemiological screening isolates, collected in UCH (Krakow, Poland) after BURP analysis with a cost of 4. Clusters of linked *spa* types correspond to *spa* clonal complexes (*spa-*CCs). The *spa* types that were defined as founders of particular clusters are indicated in blue. In total, eight *spa*-CCs have been identified, with *spa*-CC 084, *spa*-CC 002, *spa*-CC 015 and *spa*-CC 037/021/012 accounting for 14 to 41 isolates, each. Eighteen *spa* types were regarded as singletons (n = 34).

The analysis of 92 non-duplicate isolates yielded 37 *spa* types. Twelve types were shared by 2 or more isolates (n = 67, 72.8%), while 25 types were represented by a single isolate (27.2%). One new *spa* type was identified (t14391, n = 1) during this study. The most predominant *spa* types were t003, t015 and t091 accounting for 14, 13 and 11 isolates, respectively. The BURP algorithm (cost ≤4) assigned isolates to 7 *spa*-CCs (n = 73, 79.3%) and 19 singletons. Three *spa*-CCs dominated (*spa*-CC 084, n = 20; *spa*-CC 002, n = 19; *spa*-CC 015, n = 15) and accounted for 58.7% of all analysed isolates as presented in Fig 1 and additionally in S2 Table.

#### *spa* types and *spa*-CCs were associated with age of patients

As summarized in Table 2, *spa* type t003 comprised majority of *S*. *aureus* isolates in the age groups under 5 years of age demonstrating statistically significant association with invasive infections in infants and young children. Moreover, *spa* type t003 was not detected in any patient of 5 years or older. The *spa* type t2642 was obtained only from infants under 6 months of age, while type t091 was significantly underrepresented in this age group and isolated mainly from older paediatric patients. The *spa* type t008 was also more common in older children, and its association with infection increased slightly along with age (age groups of over 5 years and under 10 years). The statistical analysis of *spa*-CCs yielded results corresponding to that obtained for particular *spa* types. Thereby *spa*-CC 002 (composed of *spa* types t002, t003, t010, t088, t306) demonstrated significant association with infection in all age groups under 5 years, with all included *spa* types collected only from children between 5 days and 5 years of age. The *spa*-CC 084 (composed of *spa* types t084, t091 t360, t3850) was underrepresented in infants under 6 months of age, while *spa*-CC 008 (represented by *spa* types t008 and t1911) demonstrated association with infection in children aged 10 years or older.

#### *spa* types were stable and non-time related replacement was observed

During the study period 15 patients were sampled repeatedly and in majority of cases, the same *spa* type was identified (Table 1). The difference in time between subsequent testing ranged from 1 to 490 days (approximately 16 months). From 4 patients, two different *spa* types were collected: i) *spa* type t031 was replaced by type t091 (patient’s code AT); t091 by t003 (AY); iii) t015 by t037 (BS); iv) t14391 by t003 (CV). The interval between culture testing was 146 days, 11 days, 313 days and 21 days, respectively (Table 1). In any of those cases the two consecutive *spa* types were related according to BURP clustering results.

### DNA microarray typing

**Fig 2:**
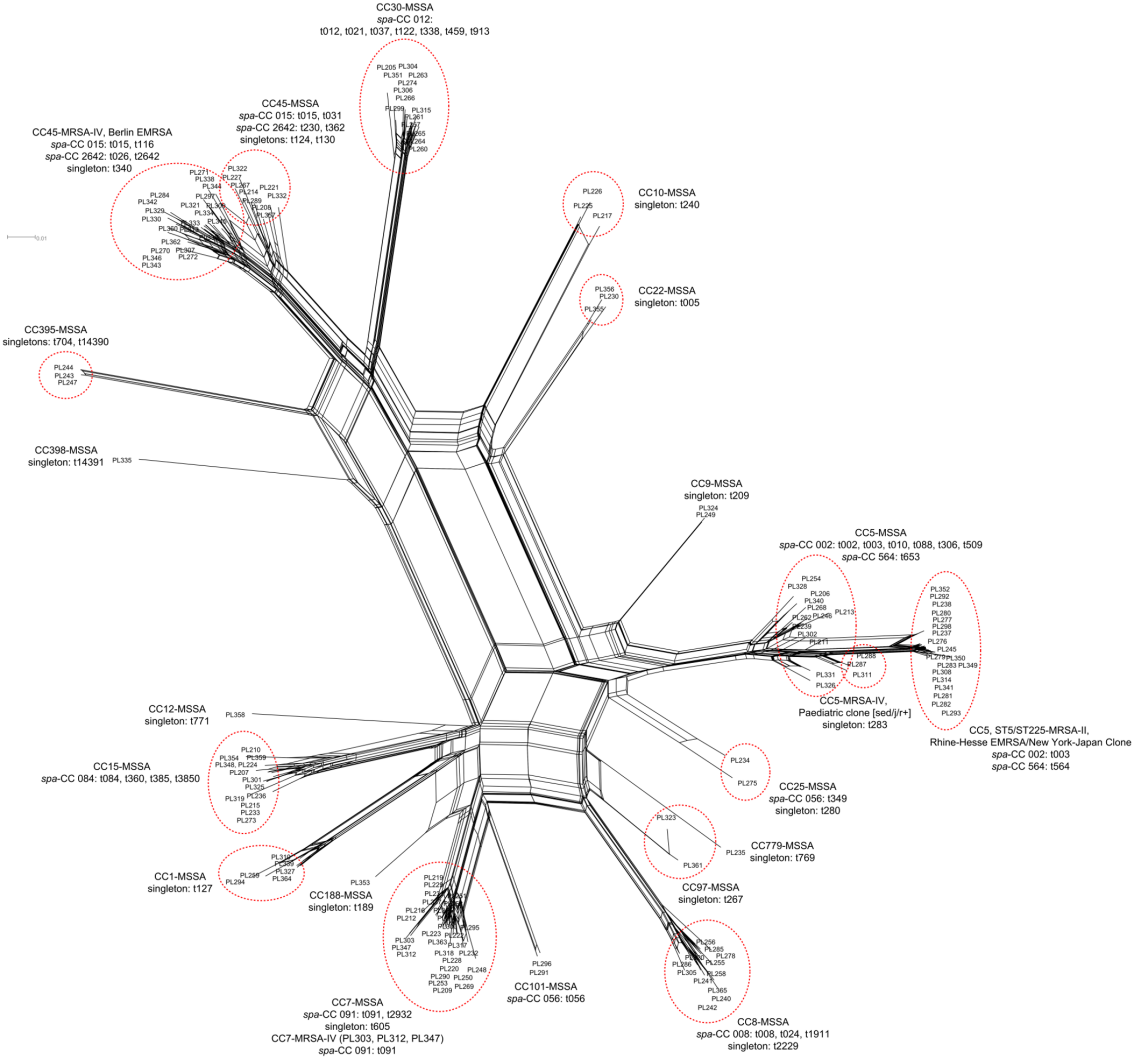
A split network tree constructed from the hybridization results of all 107 invasive *S*. *aureus* isolates from paediatric patients and 54 epidemiological screening isolates collected in UCH (Krakow, Poland). SplitsTree analysis determined 6 major (CC5, CC45, CC7, CC30, CC15, CC8) and 4 minor (CC1, CC10, CC22, CC395) clusters. There were 8 sporadic CCs represented by single/two isolates (CC9, CC25, CC101, CC97, CC779, CC12, CC188, CC398). Identified MRSA lineages included CC45-MRSA-IV (Berlin EMRSA), ST5/ST225-MRSA-II (Rhine-Hesse EMRSA/New York-Japan Clone), CC5-MRSA-IV (Paediatric clone [*sed/j/r*+]) and CC7-MRSA-IV. The *spa* types and/or *spa*-CCs are presented next to their respective CCs.

The DNA microarray-based assignment of 92 investigated isolates to CCs or STs is presented in S2 Table. A total of 8 CCs were detected. The most predominant were CC45 (n = 24), CC5 (n = 23) and CC7 (n = 11), which together constituted majority (63.0%) of analysed collection. The remaining five CCs were represented by less than 10 isolates each, which together included 34 isolates (37.0%). Affiliation to CCs and hybridization pattern similarity of all 107 isolates from current study is presented as phylogenetic network in Fig 2. All identified CCs included MSSA strains, which were highly diversified in comparison to methicillin resistant population. The MRSA were detected only in major CCs and assigned to CC45-MRSA-IV (n = 20), ST5/ST225-MRSA-II (n = 15), CC5-MRSA-IV (n = 2) or CC7-MRSA-IV (n = 1).

All isolates affiliated with CC5 were obtained from cases of invasive infection in children aged 5 or younger (Table 2). The statistical analysis yielded results corresponding to obtained for *spa* type t003 and *spa*-CC 002, which comprised majority of CC5 isolates. Therefore, CC5 was significantly associated with invasive infection children under 5 years of age. The isolates affiliated with CC8 were statistically more common in children of 10 years of age and older, similarly to results obtained for *spa* type t008 and *spa*-CC 008, also associated with infections in this age group.

#### Hybridization profile stability

During the study period, 15 paediatric patients experienced recurrent invasive infections. In the majority of cases, during subsequent isolation the same strain was identified, characterized by the same *spa* type and gene content. Obtained hybridization patterns were highly similar indicating stability of the analysed genes over time. The 34 isolates sampled from 15 patients (>2 isolates each) are listed in Table 1 and presented as phylogenetic network in Fig 2.

#### Gene content of isolates responsible for invasive infection is linked to clonal structure and subsequently age related

The results of resistance and virulence markers profiling are presented in S3 and S4 Tables.

MRSA strains are often multidrug-resistant and carriage of virulence markers is strongly linked with affiliation to clonal groups (*spa* type, *spa*-CCs, CCs).The observed in this study associations between genotype or/and methicillin resistance and patients age were reflected in gene content. The resistance determinants, *mecA*, *erm(A)* and *aadD*, were overrepresented in isolates from newborns, infants and children up to age of five, which corresponded to dominant MRSA genotype (mainly t003, *spa*-CC 002, CC5) in these age groups. Similarly, in accordance with clonal distribution, the *agr* group II (mainly t003, t283, t564 in CC5 and t084, *spa*-CC 084, CC15) were significantly more commonly found in newborns, and in children under age of five. As for the exotoxin genes, the *egc*-cluster encoding enterotoxins G, I, M, N, O and U, was more prevalent in isolates obtained from patients under age of 5 years (mainly t003, t002, t283, t564 in CC5; t015, t2642 in CC45), while enterotoxins D, J and R in infants (t003, t283 in CC5), HCA infection events and female patients. Enterotoxin A, in allelic form of strain N315 corresponding mostly to genotypes t003, *spa*-CC 002, CC5 or t091, *spa*-CC 084, CC7, was detected significantly more often in neonates. The chemotaxis inhibiting protein (CHIPS) was associated with invasive infection in infants and children under 3 years of age and was found in isolates assigned to major genotypes detected among this age group (t015, CC45; t003; CC5 or t084; CC15). All analysed isolates carried either capsular polysaccharide genes associated with capsule type 5 (*cap5*) or type 8 (*cap8*). The cap 5 alleles were significantly more common among infants, which is related with dominance of CC5 in this group. Serine protease E (*splE*) was detected mostly in MSSA isolates assigned to 8 different *spa* types and corresponding CCs. The gene was underrepresented in isolates obtained from children under 5 years of age whereas in children at age of 10 years and older, and in samples from CO infection events it was identified more frequently.

In overall, differences in carriage rates of several genetic markers were observed in MRSA and MSSA isolates collected in UCH, as summarized in S4 Table. The erythromycin or tobramycin resistance genes (*erm(A)*, *erm(C)*, *aadD*), enterotoxins D, J and R (*sed*, *sej*, *ser*), *egc*-cluster encoding several enterotoxins, staphylokinase (*sak*), chemotaxis inhibiting protein (*chp*) or fibrinogen-binding protein B (*fnbB*) were more prevalent in MRSA isolates. The toxic shock syndrome toxin (*tst-1*) and accessory gene regulator III (*agrIII*) were detected solely in MSSA isolates. Other genetic markers which were more commonly detected in this group included: haemolysin gamma C component (*lukS*), leucocidin D and E components (*lukD*, *lukE*), serine proteases A, B or E (*splA*, *splB*, *splE*).

### Emergence and distribution of *S*. *aureus* genotypes in UCH and its departments

**Fig 3:**
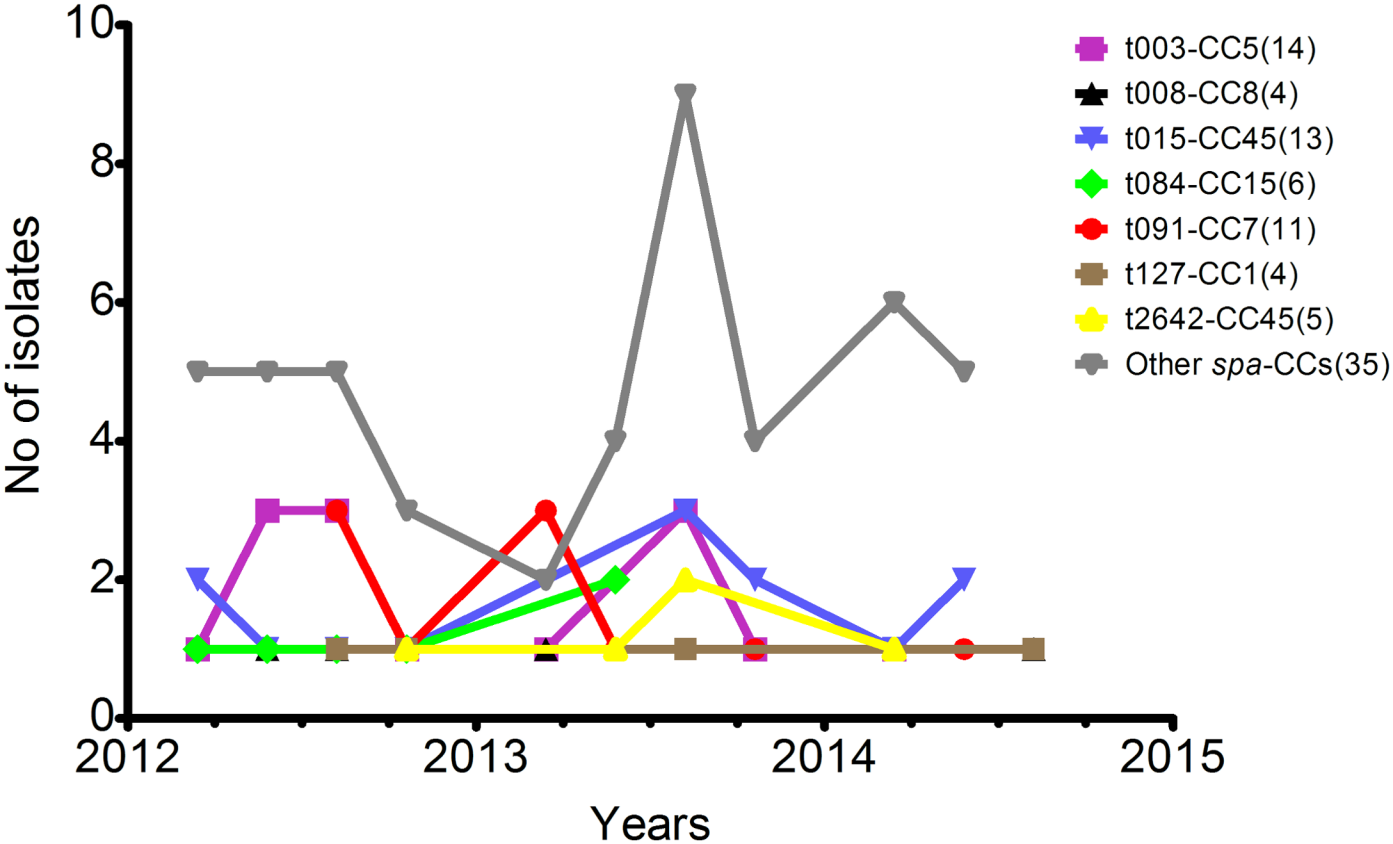
Distribution of 92 non-duplicate invasive *S*. *aureus* isolates assigned to particular *spa* type and microarray CCs genotypes collected over study duration. The numbers in parentheses indicate the total number of isolates linked with each genotype. The three dominant genotypes (t003-CC5, t015-CC45, t091-CC7) were present in UCH (Krakow, Poland) for almost entire study period, although no trend could have been observed. Remaining *spa* type-CCs (n = 35) included 2 isolates each: t267-CC97, t564-CC5, t283-CC5, t360-CC15, t002-CC5; or 1 isolate each: t056-CC101, t209-CC9, t010-CC5, t088-CC5, t005-CC22, t012-CC30, t340-CC45, t116-CC45, t1911-CC8, t122-CC30, t2229-CC8, t124-CC45, t037-CC30, t026-CC45, t306-CC5, t3850-CC15, t349-CC25. MRSA (n = 38) included following major genotypes: t003-CC5 (n = 13), t015-CC45 (n = 12), t2642-CC45 (n = 5), and 6 minor: t026-CC45, t091-CC7, t116-CC45, t283-CC5, t340-CC45, t564-CC5 with 1-2 representative isolates each.

Samples have been collected over a period of study duration from February 2012 till August 2014. The use of *spa* and DNA microarray typing allowed to determine that the most common genotypes were present in UCH for a minimum of 1 year and a half, with the following genotypes observed for the longest time periods: t015-CC45, t127-CC1 or t091-CC7. The distribution of isolates associated with particular genotypes is outlined in Fig 3.

**Fig 4:**
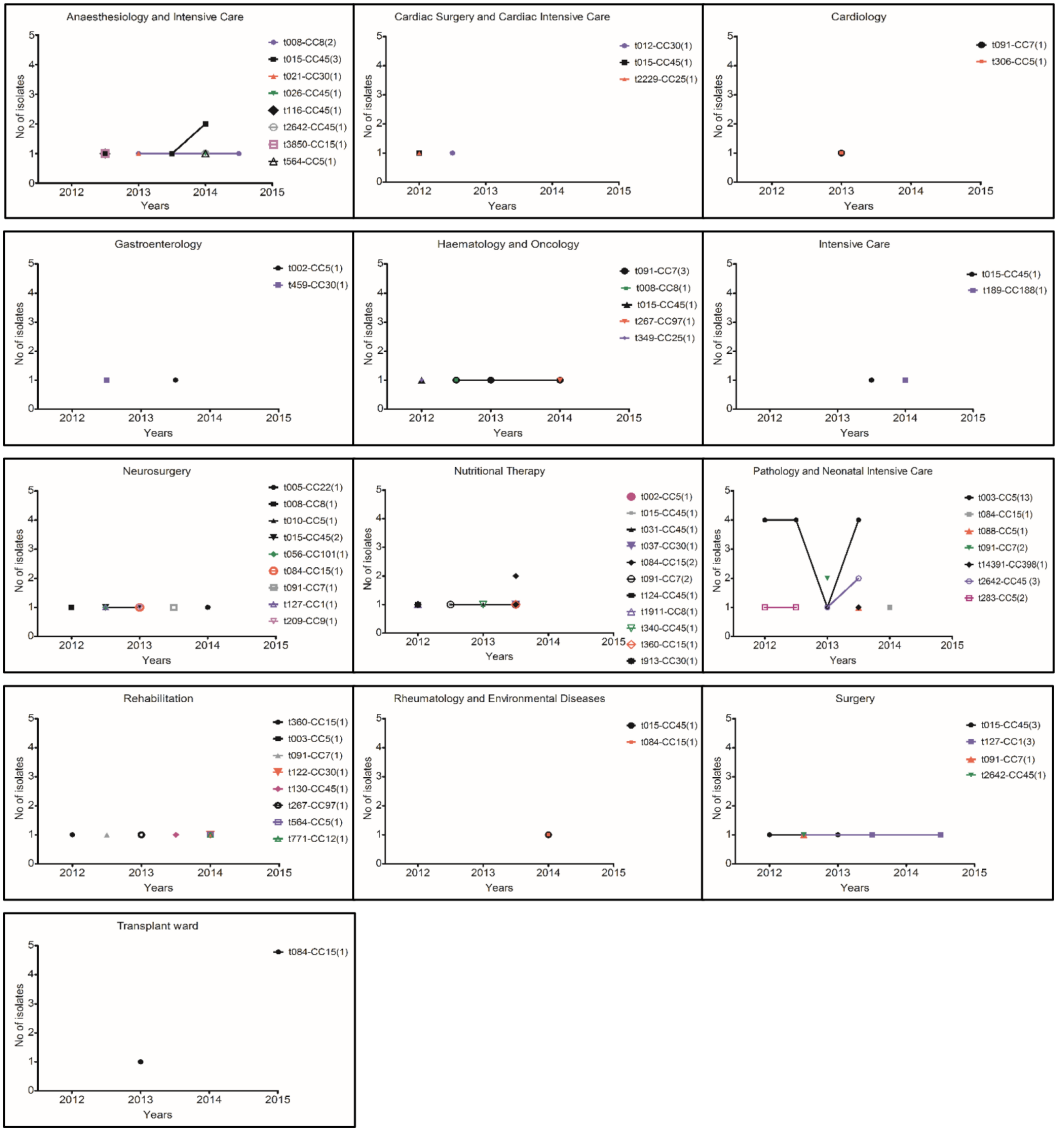
Distribution of 92 invasive *S*. *aureus* isolates in respect to *spa* types and microarray CCs genotypes collected from all examined departments of UCH (Krakow, Poland) during the study period between February 2012 and August 2014. The numbers in parentheses indicate the total number of isolates associated with each genotype (*spa* type-microarray CC). MRSA (n = 38) included following genotypes: t003-CC5 (n = 13), t015-CC45 (n = 12), t2642-CC45 (n = 5), t283-CC5 (n = 2), t564-CC5 (n = 2), t026-CC45 (n = 1), t091-CC7 (n = 1), t116-CC45 (n = 1) and t340-CC45 (n = 1).

The dominant t003-CC5 clone (n = 14), comprising mainly of MRSA identified as Rhine-Hesse epidemic line (n = 13), emerged in March 2012 and have been further detected up to November 2013 at Pathology and Neonatal IC department. Since all affected neonates were initially treated at Obstetrics and Gynaecology departments of other regional (Malopolska) hospitals, these infection events were classified as HCA Additionally, a single case of MSSA-t003-CC5 isolate, also considered as HCA, was identified in Rehabilitation department in 2014. The second most common genotype, t015-CC45 (n = 13), was found in 12 isolates and is assigned to Berlin-EMRSA clone. One MSSA isolate was detected in Surgery department. The t015-CC45-Berlin EMRSA clone was firstly encountered in February 2012 in CO infection event at Haematology and Oncology department and was subsequently isolated in samples from both CO and HCA infections occurring till June 2014 in in seven other UCH units. The t091-CC7 clone (n = 12, including 11 MSSA) was initially identified in Nutritional Therapy department and became a cause of CO or HCA infections in other seven different departments between October 2013 and June 2014. The MSSA clone t084-CC15 (n = 6) also emerged in Nutritional Therapy and was further identified in HCA or CO infection events in overall five UCH departments between August 2013 and April 2014. Genotype t2642-CC45 (n = 5), also assigned to Berlin-EMRSA epidemic line, was firstly detected in Surgery department in October 2012, later in 2013 in Pathology and Neonatal IC and in 2014 in Anaesthesiology and IC department. All its cases were regarded as HCA. MSSA clone t008-CC8 (n = 4) was identified in three HCA and one CO infection between June 2012 and August 2014, initially in Neurosurgery and later in two other UCH departments. All isolates affiliated to t127-CC1 (n = 4) were MSSA and caused HCA invasive infections occurring firstly in Neurosurgery and later in Surgery departments in period from August 2012 till July 2014. The details on remaining 30 genotypes detected in UCH (n = 35) are provided in S2 Table and additionally in Fig 4 presenting distribution of isolates collected from different UCH departments in respect to particular *spa* type-microarray CCs.

Among the examined 13 UCH departments, Pathology and Neonatal IC Unit characterised with significantly higher number of MRSA, HCA infection events and incidence of isolates assigned to CC5, especially t003-CC5 genotype known as Rhine-Hesse epidemic line, when compared to other UCH departments (p < 0.05). Interestingly, in the same department t015-CC45 clone (mostly MRSA) was underrepresented and not detected among overall seven genotypes identified (Fig 4). Due to the unit specificity, all tested isolates were collected from neonates or infants. Consequently, patients under 1 year of age and younger were overrepresented when compared with other departments (Table 2). As depicted in Fig 4, the highest number of different *S*. *aureus*, mostly MSSA, was observed in the department of Nutritional Therapy which also admitted significantly lower proportions of neonate or infant patients (Table 2). Among the overall eight *spa* types identified in Anaesthesiology and IC department, four were affiliated to CC45 (t015, t026, t116, t2642) which translates into the overrepresentation of that particular CC when compared to other isolation sites (p < 0.05). Significant majority of infection events investigated in the department of Haematology and Oncology was classified as CO (p< = 5), and occurred more often in children over 5 years of age as summarized in Table 2. The genotype t091-CC7 was prevailed in this department (3 of 7 isolates total) and was also detected in other six UCH departments. Children from Rehabilitation department with invasive infections were in significant majority older than 5 years of age (Table 2), and each of eight obtained infection isolates collected between 2012 and 2014 was associated with different genotype (Fig 4). Similar genetic diversity was observed in Neurosurgery department, as among 9 genotypes identified only t015-CC45 was isolated more than once Fig 4). Although t127-CC1 genotype initially observed in Neurosurgery unit, it was later detected and overrepresented in Surgery department (p < 0.05).

### Epidemiological screening results

Complete information and genotyping results for 54 *S*. *aureus* isolates obtained during epidemiological screening are provided in S1 and S5 Tables, respectively. Overall, 27 *spa* types and 13 CCs (based on microarray analysis) were identified, with the highest prevalence of t091-CC7 (n = 13), t037-CC30 (n = 5), t008-CC8 (n = 4) and t240-CC10 (n = 3) genotypes. The single case of t003-CC5 isolate (PL245) was identified as MRSA carriage and with the use of StaphyType software was assigned to Rhine-Hesse epidemic line. The screening process of patients (n = 7) revealed the presence of six genotypes t002-CC5, t088-CC5, t091-CC7, t230-CC45, t360-CC15 and t509-CC5. With exception of t230-CC45, the aforementioned genotypes were also detected in isolates from invasive infection cases collected during the study period. Three patients admitted to Nutritional Therapy department were tested both for invasive infection and *S*. *aureus* carriage (patients’ codes: AT, AW and CA; S1 Table). In May 2013, following the onset of invasive infection, patient AT was tested and positive for t031-CC45 isolate (PL322). However, in October 2013 during a second onset of infection, t091-CC7 genotype was isolated (PL209) which was also collected week later from nasal swab during epidemiological screening (PL212). In case of patient AW, the isolate from invasive infection occurring in August 2013 revealed the presence of t084-CC15 isolate (PL207). Later in October 2013 the t002-CC5 genotype was identified in nasal swab (PL211). Similarly, patient CA was initially a carrier of t002-CC5 genotype (PL213) as tested in October 2013, but the invasive infection later in December 2013 (PL236) and January 2014 (PL348) was caused by t084-CC15.

Among 36 isolates collected from medical personnel, t091-CC7 (n = 5), t037-CC30 (n = 4) and t008-CC8 (n = 4) dominated, while 20 other *spa* types and their corresponding CCs were identified in 23 remaining isolates (S5 Table). Fifteen, 11 and 10 isolates were obtained from personnel working in Cardiac Surgery and Cardiac IC, Nutritional Therapy and Pathology and Neonatal IC departments, respectively. In case of four personnel members encoded as ER, EW, EX and FC, from which nasal swabs and hand smears/fingerprints on agar substrate were retrieved, identical genotype was detected in both tested materials. As sample obtained in September 2013 suggests, one member of personnel was a carrier of t003-CC5-Rhine-Hesse MRSA epidemic line (PL245) around that time. This epidemic clone emerged in UCH over year earlier in March 2012. It was overrepresented and prevailed in Pathology and Neonatal IC department during the study period. Other genotypes which were detected both in invasive infection cases and as carriage by medical personnel were t091-CC7, t015-CC45, t008-CC8, t037-CC30, t127-CC1, t360-CC15, t002-CC5, t005-CC5, t509-CC5, t012-CC30 and t209-CC9 (S1 Table).

The epidemiological screening of electronical or medical equipment, furniture, toys, door handles or air yielded positive results for *S*. *aureus* presence (n = 11, mainly in Nutritional Therapy department, n = 9). Majority of examined *S*. *aureus*-positive isolates was related to t091-CC7 clone (n = 7), although four more genotypes were detected, t037-CC30, t124-CC45, t240-CC10 or t653-CC5, with single representative isolate each. The t240-CC10 and t653-CC5 were identified to be carried by personnel or on equipment, but were not observed in invasive infection cases (S1 Table).

The t091-CC7 was the only genotype detected in invasive infection cases (n = 14, including 3 MRSA) and in screening samples from patient (n = 1), medical personnel (n = 5) or UCH equipment (n = 7, S1 Table).

## Discussion

*Staphylococcus aureus* is one of the most common opportunistic pathogens in adult and paediatric populations acquired in both community or hospital settings [3,7,18–20]. The current study aimed to analyse *S*. *aureus* genotype, including virulence and resistance gene carriage, with regard to age of children treated in UCH of Krakow, Poland. Approximately half of 107 analysed *S*. *aureus* isolates were collected from patients experiencing invasive infection at 1 year of age or younger, including neonates who developed symptoms between 5th and 28th day after birth. The total of 88 paediatric patients originating from vast geographic region (45 300 km^2^) were admitted and arrived directly from home or were transferred to UCH from other healthcare facilities. Due to highly specialized profiles of UCH clinics, the patients were exposed to wide range of multi resistant pathogens. From the majority of cases one isolate was collected, whereas 15 patients were repeatedly tested and positive to be infected (or re-infected) with the same strain or various strains. Additionally, 54 epidemiological screening *S*. *aureus* isolates were retrieved and analysed to determine and track genotypes among non-invasive infection patients, medical personnel, equipment and hospital spaces.

Majority of infection events which yielded the samples for this analysis were assessed as HCA and occurred in population of infants and children not older than 5 years. These findings correspond to previous reports from other countries. In Spain, in children with bloodstream infections HCA *S*. *aureus* accounted for 77.2% episodes [21]. Further sources provide statistics of similar magnitude. The Canadian report states that all *S*. *aureus*-related infections in neonates were of HCA type [6]. In UK approximately two thirds of MSSA bacteraemia were acquired in healthcare setting [22]. Also in this study, the presence of MRSA isolates of HCA origin was detected most frequently in population of children aged 5 years or less. On the other hand, the MSSA strains of CO origin were significantly more common among patients aged 5 years—10 years and older. However, in contrast to European and worldwide data, in present set of samples only five have been obtained from infection episodes related to MRSA of CO origin [23–26].

Considering the risk of death due to *S*. *aureus* infection, recently an association has been found between methicillin resistance and higher mortality rate in neonates and children diagnosed with *S*. *aureus* bacteraemia [27]. In the current study four fatal cases have been reported and three of them were tested positive for MRSA in blood samples. The observed all-cause mortality and 14 day all-cause mortality were comparable with previously reported in children, but much lower than estimated for general population [6,12]. Study design and methodological restraints did not allow to assess the impact of infection on health outcomes in patients.

In 15 patients, blood samples were collected several times at different time points. Eleven patients were identified to be infected with strains of the same *spa* type and highly stable DNA microarray profiles, as observed differences were of allelic or single locus nature. The remaining four children had the same *spa* type detected in the first episode/s, but a different type in the last analysed sample. The similar observation has been previously reported and postulated as a tendency of patients to experience recurrent infections of initial infecting strain [22]. As no clinical data were available, we could not determine whether the multiple testing was performed as a part of therapy/monitoring or due to the episodes of re-infection.

The age-dependent incidence of *S*. *aureus* infection is widely documented. In two recent studies, one multinational and one based on Danish medical database, the rates of bloodstream infection are high in children under 1 year of age, drop significantly in older age groups, and rise to equal and higher levels above age of sixty [28,29]. In a population based study from Canada, the risk of developing *S*. *aureus* bacteraemia was highest in neonatal period and fell significantly after 1 month of age and later childhood [6]. Yet, the association between *S*. *aureus* genotype and age of the patient have been investigated only to a small extent. For instance, the particular *spa* types have been associated with age of carriers in general population, moreover CC5 and CC45 were reported to exhibit age preferences for carriage or infection in adults [30,31].

In this study, in line with other European reports on genotyping, the types t003, t015, t091, t084, t2642, t008, t127 and CCs: CC45, CC5 and CC7 dominated among the overall 51 *spa* types and 18 CCs identified among all 161 tested isolates [12,21,31–34]. Some of the observed major or minor genotypes exhibited age-related pattern of infection among patients treated in UCH. The *spa* type t003, as well as *spa*-CC 002 and CC5 were strongly associated with invasive infections in infants and young children. Additionally, these genotypes were the most common among MRSA infections of HCA origin to be found in patients aged 5 years or less. Recently, the t015-SCC*mec* type IV strains were identified as the most common cause of MRSA infections in Polish newborns [2]. However, in the current study no age relatedness was observed for t015 (*spa*-CC 015, CC45), which was the second major MRSA genotype in UCH. In general, the predominance of *spa* types t003 and t015 among MRSA and HCA isolates is well documented. Both types are reported to be second in prevalence among MRSA *spa* types in Poland and to frequently cause HCA-MRSA infections in Europe [12,30,35–37]. The t2642 (singleton, CC45) was overrepresented in children under 5 years of age and not identified in older patients. This *spa* type was not reported previously to infect children, but its presence was found in Europe and detected in both MRSA and MSSA isolates as confirmed in the Ridom *spa* server (http://spa.ridom.de/spa-t2642.shtml). The *spa* type t091 was underrepresented in infants under 6 months of age and isolated mostly from older infants or children treated in UCH. Similarly, the *spa* type t008 along with *spa*-CC 008 and CC8 were most commonly obtained from patients above age of 5 or 10 years. Observed frequencies of t091 (CC7) and t008 (CC8) are in accordance with previous reports, as this genotype is the most common MSSA clone in Europe [37]. The *spa* type t008 was previously reported as one of the most common in paediatric MRSA-related infections in USA, although in the current study it was associated with MSSA infections [38].

Numerous attempts have been made to assess the virulence gene carriage in *S*. *aureus* bacteria, CCs or even particular epidemic lines. According to findings of molecular genetics, the host specificity of particular *S*. *aureus* lineages is reflected in strain genetic profile, through presence or absence of allelic variations or insertions. Consequently, the observed gene frequencies are complementary to genotyping data, resulting from number and proportions of particular genotypes within analysed subgroups. In the current study, carriage of several genetic markers was significantly higher among particular age groups, including *egc-cluster*, *sej/sed/ser*, *chp* or *splE*. The results of recent murine model experiments, suggest that *egc*-cluster (*seg*, *seln*, *selu*, *sei*, *selm*, *selo*), located on vSaβ genomic island, only marginally contributes to virulence but might be involved in colonization of mucosal surfaces [39]. In the current study *egc*-cluster was overrepresented among MRSA isolates (CC5, CC45), and similarly to CC5 genotype highly prevalent in children under 5 years of age. In Ireland, the high frequencies of this MGE have been reported for MRSA strains [40]. However, the cluster was also detected in several isolates lacking *mecA* gene, affiliated mostly to CC30, CC5, CC45 or other minor CCs, corresponding to previous reports for MSSA infection [31,41,42].

In UCH isolates, the plasmid encoded enterotoxins D, J and R (*sed+sej+ser*) were overrepresented in categories of MRSA isolates, children under 5 years old or female patients. Generally, the enterotoxins D, J and R are common in clinical *S*. *aureus* isolates [36,43,44]. The *sed+sej+ser* genes were detected in isolates of CC5 (both MRSA and MSSA) and CC8 (MSSA), and most likely carriage within CC5 resulted in observed high frequencies in the analysed subgroups. The *sed* gene was earlier reported to be significantly more frequent among invasive MRSA isolates [45,46]. Although the presence of enterotoxins in paediatric isolates from bloodstream infections was previously reported in China, the dependency on age or sex was not analysed, and current findings will require further investigation [11].

*S*. *aureus* bacteria produce factors responsible for evasion and modulation of the innate host immune response [47,48]. The immune-evasion cluster (IEC) genes *sak*, *chp* and *scn*, encoding staphylokinase, SCIN and CHIPS, respectively, are transmitted together by several staphylococcal bacteriophages, integrating within *hlb* gene [47,49,50]. Although UCH isolates carrying *chp* and *sak*, were nearly equally divided between MRSA and MSSA subgroups, both genes were overrepresented among MRSA (CC5 and CC45). Similarly, high frequencies of IEC genes were also observed among MRSA isolates in Ireland [40], although in Romania when characteristics of MRSA and MSSA isolates were compared no differences were observed [51]. The CHIPS was recently connected with nasal colonization of young children, which might partially explain identified here age factor, as the gene was present in both MRSA and MSSA isolates [47,52].

The *splE* encoding serine protease E is frequently reported to be present in clinical *S*. *aureus* strains and in this study was the only gene overrepresented in isolates collected from older children [33,51,53]. This observation corresponds to higher rates of MSSA infection among UCH patients over 5 or 10 years, as the *splE* was carried by several MSSA-CCs, mainly CC7, CC15, CC8 or CC30. The age-related patterns of this determinant have not been reported previously. The comparison between carriage and invasive isolates in Sweden showed no statistical differences [33].

Beside determinants discussed earlier, also some other resistance or virulence markers were unevenly distributed between MRSA and MSSA isolates. In accordance with worldwide reports, the MRSA isolates were characterized by significantly higher rates of genes mediating resistance to MLS-antibiotics (*erm(A)/(C)*) or tobramycin/aminoglycosides (*aadD*) when compared to MSSA-related values [40,44,54–56]. In the current study, the *agr* type III was overrepresented among MSSA isolates, affiliated with CC1 or CC30, which corresponds to results from other population based studies [16,56]. The observed rate of TSST-1 (*tst-1*), associated with staphylococcal toxic shock syndrome, was much lower than reported in Norway, Germany, Spain or other European countries [9,31,35,57,58]. The carriage rates of *tst-1* were significantly higher in MSSA isolates. The lack of TSST-1 in MRSA subgroup might have resulted from SaPI-mediated transmission of the gene [50,59,60]. Additionally, the distribution of TSST-1 was reported to follow CCs affiliation, and, indeed, the *tst-1* gene was detected mainly in CC30 [16,61,62]. Similarly, the serine proteases A and B (SplA, SplB) were significantly more common in MSSA isolates. This observation corresponds to previous reports, as those markers are often detected in clinical isolates in countries with low incidence of MRSA [32,33,51,56]. Serine protease A and B are especially common in invasive *S*. *aureus* infections or atopic dermatitis [33,63]. In the current study the *lukS* gene encoding γ-haemolysin C component was overrepresented in MSSA isolates, while in a recent report from Ukraine no differences were observed between MRSA and MSSA [64]. Similarly, although the leukocidin components D and E are reported to be prevalent in MRSA clones, in the analysed collection *lukE/D* genes were more frequent in MSSA isolates [16,51,65] Those discrepancies might have resulted from differences in *S*. *aureus* clonal distribution in Europe, and *lukS/E/D* gene association with MSSA infection requires further verification. Fibrinogen-binding protein, encoded by *fib* gene, was found significantly more often in MSSA isolates, which corresponds to high rates of this gene reported in Sweden [56,58]. However, in a recent study comparing MSCRAMMs carriage rates in paediatric isolates, no differences in *fib* gene frequencies were observed in MRSA and MSSA groups [66]. Fibronectin-binding protein B (*fnbB*) was present in all analysed MRSA isolates and consequently overrepresented in this group, even with 85% rate in MSSA isolates. In general, the *fnbB* is usually present in CC5, which in the current study was one of two major clones responsible for MRSA infection among UCH patients [16].

Based on analysis results of *S*. *aureus* isolates retrieved from invasive infection cases and during epidemiological screening, the major identified clones have been present in UCH since year 2012, and circulating between patients, medical personnel, hospital spaces or equipment. With few exceptions, identical genotypes have been detected in samples from invasive infection episodes and screening, with t091-CC7 being most ubiquitous. Four distinct MRSA lineages were found, with t003-CC5 clone (ST5/ST225-MRSA-II) known as ‘Rhine-Hesse EMRSA’ being predominant among invasive infection isolates from neonates or infants. Previously, Rhine-Hesse isolates were classified as HCA and reported in Germany and Austria since year 1995 [16,67]. The most prevalent MRSA clone in UCH, CC45-MRSA-IV (Berlin EMRSA), was firstly observed in Berlin hospitals in 1993 and later spread within healthcare institutions all over Germany, the Netherlands and other European countries [68–71]. Similarly, widely reported lineage CC5-MRSA-IV (Paediatric clone [*sed/j/r+*]) most likely originated in Portugal, being firstly described in paediatric hospital in 1999 [68,72,73]. Detection of above MRSA epidemic lineages in South Poland paediatric hospital further affirms existence of international transmission of HCA-associated clones described previously in European structured surveys [12,37]. Among the examined departments, the Pathology and Neonatal IC Unit showed to have significantly higher rates of HCA-MRSA. This highly specialised department of UCH is dedicated for diagnosis and treatment of preterm infants, children with congenital defects, inborn conditions, requiring mechanical ventilation and/or with symptoms of bacteraemia/sepsis. Among paediatric patients tested and positive for *S*. *aureus* invasive infection treated in this department, 16 were admitted from other hospitals in Malopolska (mostly from maternity or neonatal units) and were source of 17 isolates as summarised in S1 Table. Five children were admitted directly from home and in case patients BC (PL292) and BV (PL298) the admissions were classified as emergency. Patient BJ was initially admitted to Cardiac Surgery and Cardiac IC department but was later transferred to Pathology and Neonatal IC within UCH, where he was tested and positive for *S*. *aureus* invasive infection (PL277, S1 Table). The emergence and predominance of t003-CC5 genotype (including Rhine-Hesse epidemic line) in above UCH unit, might be associated with significantly younger age of admitted patients and IC profile of this department. It has been previously reported that invasive MRSA infections mostly occur in children younger than 1 year of age, and presence of comorbidities, invasive devices, exposure to broad-spectrum antibiotic or parenteral nutrition increase risk of infection [1,74–76]. The t003-CC5 (Rhine-Hesse epidemic line) was also the only MRSA clone detected in nasal swab sample obtained from one member of medical personnel. Subsequently, the observed MRSA carriage rate among UCH personnel (3.1%) was below worldwide estimate and results reported in other studies [77–79]. Overall, genotypes identified during epidemiological screening belonged to similar genetic lineages as those collected from patients with invasive *S*. *aureus* infection. The existence of an active transmission between patients, personnel and by contact with medical/other equipment is highly suggestive, although its direction might not be only towards the patients, but also *vice versa*. As reported recently, based on Whole-Genome Sequencing (WGS) data gathered during MRSA outbreak, tracing evolutional changes in *S*. *aureus* genomes suggests existence of much more complicated transmission patterns than anticipated before introduction of WGS technology [80].

In, conclusion, invasive *S*. *aureus* infections, including bacteraemia or sepsis, are one of the most fatal bacterial diseases worldwide. Both in immunocompromised or previously healthy children, *S*. *aureus* is found to be a cause of approximately 10% of bloodstream or invasive infections [3,20,81]. In the current study, high diversity of MSSA strains was observed, whereas MRSA infection was of clonal nature, with few dominant lineages. The *spa* types predominant in UCH of Krakow, Poland were similar to those reported in other European studies. The isolates retrieved during epidemiological screening were mostly MSSA and belonged to similar genetic lineages as those collected from patients with invasive infection. The association between *S*. *aureus* genotype and age of patients was identified and shown in the population of children under 5 years of age. The observed in UCH associations between genotype (*spa* types, CC, genetic profile) and patients characteristics, might reflect microorganism and host matching, where gene content of both pathogen and patient interact during colonization and pathogenesis. However, the observed strain diversity indicate that host factors might be at least as important as bacterial genetic profile during pathogenesis process explaining high incidence of *S*. *aureus* invasive infections in children and adults. Our results ought to be further investigated in the multiple-center settings in paediatric population to address the high incidence and burden of *S*. *aureus* infections in infants and children.

## Supporting Information

S1 Table Full information on 107 invasive infection and 54 epidemiological screening *S*. *aureus* isolates collected in UCH (Krakow, Poland). Isolates included in statistical and epidemiological analysis are highlighted in grey. Isolates collected during epidemiological screening are highlighted in orange. To protect patients and medical personnel identity an alphabetic code has been assigned to each, regardless of their names or initials. The presented data are arranged chronologically. CSF- cerebrospinal fluid; DF—dialysis fluid; CC—clonal complex; CO—community-onset; HCA—healthcare-associated; S—susceptible; R—resistant; MRSA—methicillin resistant *S*. *aureus*; MSSA—methicillin susceptible *S*. *aureus*; MLS_B_—macrolide-lincosamide-streptogramin B (resistance); N/A—not applicable; nd—no data.
(XLSX)

S2 Table Classification and molecular typing data on 92 non-duplicate invasive *S*. *aureus* isolates obtained from 88 paediatric patients treated in UCH (Krakow, Poland). CC—clonal complex; CO—community-onset; HCA—healthcare-associated; MRSA—methicillin resistant *S*. *aureus*; MSSA—methicillin susceptible *S*. *aureus*; MLS_B_—macrolide-lincosamide-streptogramin B (resistance).
(XLSX)

S3 Table Identified statistically significant associations between age of paediatric patients and carriage of gene markers among invasive *S*. *aureus* isolates collected in UCH (Krakow, Poland). The grey background indicates that given category is overrepresented in analysed age group.
(XLSX)

S4 Table Identified statistically significant associations between HCA/CO infection event/gender/methicillin resistance and carriage of gene markers among invasive*S*. *aureus* isolates collected in UCH (Krakow, Poland). The grey background indicates that given category is overrepresented in analysed age group. CO—community-onset; HCA—healthcare-associated; M—male; F—female; MRSA—methicillin resistant *S*. *aureus*; MSSA—methicillin susceptible *S*. *aureus*.
(XLSX)

S5 Table Classification and molecular typing data on 54 *S*. *aureus* isolates collected during epidemiological screening in UCH between September and October 2013 (Krakow, Poland). Isolates collected during epidemiological screening are highlighted in orange. To protect patients and medical personnel identity an alphabetic code has been assigned to each, regardless of their names or initials. The presented data are arranged chronologically. CC—clonal complex; CO—community-onset; HCA—healthcare-associated; S—susceptible; R—resistant MRSA—methicillin resistant *S*. *aureus*; MSSA—methicillin susceptible *S*. *aureus*; MLS_B_—macrolide-lincosamide-streptogramin B (resistance); N/A—not applicable; nd—no data.
(XLSX)
