# Correction: Clonal Structure and Characterization of *Staphylococcus aureus* Strains from Invasive Infections in Paediatric Patients from South Poland: Association between Age, *spa* Types, Clonal Complexes, and Genetic Markers

**DOI:** 10.1371/journal.pone.0154485

**Published:** 2016-04-21

**Authors:** The PLOS ONE Staff

The following information is missing from the Funding section: This work was supported by the Interreg IVa-funded projects EurSafety Health-net (III-1-02 = 73) as part of a Dutch-German cross-border network supported by the European Commission, the German Federal States of Nordrhein-Westfalen and Niedersachsen, and the Dutch provinces of Overijssel Gelderland Limburg.

The publisher apologizes for the error.
